# Retraction of: Causal relationship between multiparameter brain MRI phenotypes and age: evidence from Mendelian randomization

**DOI:** 10.1093/braincomms/fcae461

**Published:** 2025-01-07

This is a retraction of: Xinghao Wang, Qian Chen, Yawen Liu, Jing Sun, Jia Li, Pengfei Zhao, Linkun Cai, Wenjuan Liu, Zhenghan Yang, Zhenchang Wang, Han Lv, Causal relationship between multiparameter brain MRI phenotypes and age: evidence from Mendelian randomization, *Brain Communications*, Volume 6, Issue 2, 2024, fcae077, https://doi.org/10.1093/braincomms/fcae077

After publication of this article an independent expert in the field contacted the Journal with concerns over analyses used in the study, specifically around causal inference from genes to age. These concerns were not picked up by the peer reviewers of the paper in two rounds of external review. However, the Editor in Chief believed they were serious enough to warrant further investigation and consulted further experts who shared these concerns. Although the authors were keen to work with the Journal to correct the paper, the experts consulted concluded that the authors’ proposed correction of the published work clarifying that “age at death” instead of “age” was used in the analyses would be insufficient to address all concerns raised. Therefore, the editors are retracting the article. This retraction is not due to data falsification or misconduct.

